# Synthesis of methylprednisolone loaded ibuprofen modified dextran based nanoparticles and their application for drug delivery in acute spinal cord injury

**DOI:** 10.18632/oncotarget.20649

**Published:** 2017-09-05

**Authors:** Lei Qi, Haiyan Jiang, Xiaohui Cui, Guiwen Liang, Ming Gao, Zhongwei Huang, Qinghua Xi

**Affiliations:** ^1^ Department of Emergency Medicine, Affiliated Hospital of Nantong University, Jiangsu, China; ^2^ Department of Geriatric Medicine, Affiliated Hospital of Nantong University, Jiangsu, China; ^3^ Jiangsu Key Laboratory of Neuroregeneration, Nantong University, Nantong, China; ^4^ Department of Obstetrics and Gynecology, Affiliated Hospital of Nantong University, Jiangsu, China

**Keywords:** methylprednisolone, nanoparticle, drug delivery, drug nanocarrier, acute spinal cord injury

## Abstract

To improve the therapeutic efficacy of spinal cord injury (SCI), the methylprednisolone was incorporated into nanoparticles based on the ibuprofen modified dextran. The ibuprofen modified dextran was synthesized using a direct esterification linkage between the carboxylic acids of hydrophobic drug and the hydroxyl groups of the polymer backbone. The morphology of methylprednisolone loaded nanoparticles was evaluated by transmission electron microscopy (TEM) and dynamic light scattering (DLS). The therapeutic efficacy of the prepared nanoparticles on the acute SCI model rats was assessed. It is demonstrated that methylprednisolone loaded ibuprofen modified dextran based nanoparticles (MP-loaded NPs) could promote the recovery of neurological deficits, enhance growth of neurons, decrease degeneration of injuried neurons and reduce the tissue tumor necrosis factor alpha (TNF-α) levels significantly in the SCI rats. Subsequently, the study indicates that synthesis of methylprednisolone loaded ibuprofen modified dextran based nanoparticles has a great potential in the synergetic effect treatment for spinal cord injury and nanoparticles based drug delivery system will become a powerful weapon of human conquest of disease.

## INTRODUCTION

Spinal cord injury (SCI) has become one of the worldwide clinical problems, which can negatively affect both the patients and the health care system [1]. Traumatic SCI initiates a cascade of cellular and biochemical events, which lead to devastating sensory and motor functional impairment, neurological deficits, and permanent paralysis, involving disruption of tissue integrity, blood vessel and axon injuries, edema and cell membrane damage [2]. Primary injury is the neurological damage at the time of insult. Following the primary injury, secondary damage happens with biochemical changes which cause free radical damage as well as the additional deterioration of the original area caused by lipid peroxidation [3]. The most important mechanisms that cause neuronal damage after SCI are inflammation, lipid peroxidation and oxidative stress [4]. The pathophysiologic and biochemical changes after SCI have been investigated to develop the treatments which may minimize function loss [3].

Although much effort has been tried, there is still no effective treatment for SCI and the recovery process remains inefficient. Although many therapeutic methods and agents including many neuroprotective drugs have been investigated to minimize injury to prevent the secondary damage after the experimental injury, only methylprednisolone (MP) has been shown to provide benefit in practice currently [5]. Systemic high-dose administration of MP could minimise the losses of the human neurological deficits after SCI. Although the latent therapeutic mechanism of MP was unclear, people believed that the primary therapeutic effect after SCI might be relevant to the prohibition of lipid peroxidation and inflammatory response [6]. Unfortunately, high-dose systemic MP used in the SCI could lead to serious side effects, such as gastric bleeding, sepsis, pneumonia, acute corticosteroid myopathy and wound infection, which could accompany only with modest improvements in the neurological recovery [7]. The side effects of MP therapy as just mentioned may be related to the high systemic dosage and its toxicity. Therefore, the targeted delivery of MP to the injury site is likely the major obstacle to the effectively and widespread clinical use of MP [6].

Recently, increasing interest and intensive effort have been devoted to biomedical used drug delivery carriers in nanoparticles (NPs)[8]. Nanoparticles based drug delivery system could easily permeate deeply into tissues and fine capillaries because of their sub-cellular and sub-micron size. In particular, natural polysaccharides based bio-nanoparticles, are widely used for drug delivery systems, such as anti-inflammatory drugs [9], antibiotics [10], proteins [11], gene [12], peptides [13] and hormones [14], all due to their remarkable superiority in biodegradability and biocompatibility [15]. On the other hand, suitable modification of polysaccharides allows for an improvement in the properties of natural polymers [16]. For example, These modified polysaccharides could self-assemble into nanoparticles with the hydrophobic segments forming the core and hydrophilic segments forming the shell in the aqueous solution [17]. These polysaccharides based nano-drug delivery systems have significant advantages, such as stabilizing the therapeutic agents, improving the solubility of hydrophobic drugs, prolonging the circulation life-time and reducing the side effects of the drugs [16, 17].

Dextran is a branch of polysaccharide, often used to cover nanoparticles by being obtained by microbiological composition [18]. The bacterial strainwith with varying branches, which can produce dextran, composes dextran with α-(1→6) and partly α-(1→3) linked D-glucose units [19]. It has been widely used clinically, including peripheral flow promotion, plasma volume expansion, some antithrombolytic agents, and so on [20–22]. As the drug-delivery systems with no surface charges could increase the rate of nonspecific cellular uptake and reduce plasma protein adsorption, Dextran has no surface charges, which leads to additional advantages for the drug-delivery system [23]. Because of its nontoxicity, biodegradability, and hydrophilicity, Dextran may promote the intracellular absorbtion of dextran covered with magnetic iron particles [24]. Due to the presence of high amount of hydroxyl groups facilitating the introduction of drugs into the polymer backbone, Dextran has been used to delivery various pharmaceutical agents as efficient prodrugs, such as mitomycin C [25], daunorubicin [26], and naproxen [27]. Recently, the use of a dextran-based nanosystem for delivery of doxorubicin and small interfering RNA in tumor cells has been reported, indicating that Dextran plays a potential drug-delivery proficiency [25, 28].

Generally, the polymer includes linear chains of fructose unit chains linked by glycosidic bonds and ended at a single glucopyranoside ring. Due to its biodegradability and biocompatibility, Dextran is a good candidate for biomedical applications including tissue engineering and biodegradable drug-loaded pariticles [28]. Preparing the copolymers of hydrophobic drug and polysaccharides would be a significant progress in the area of drug-delivery system. Up to now, some polysaccharides graft copolymers have been already successfully prepared, and they could be potentially used for the controlled released systems and other biomaterials applications [29].

In our study, it is aimed to develop a nanoparticle based drug delivery system of MP and to study its application for SCI. In present work, we report a synthetic method of MP loaded nanoparticles, specifically, combining the non-steroidal anti-inflammatory drug ibuprofen and decorated Dextran nanoparticles into a new nanoparticle based drug delivery system. The nanoparticle was prepared by a direct esterification linkage between the hydroxyl groups of Dextran and the carboxylic acid groups of ibuprofen with the help of N, N-carbonyldiimidazole which in-situ activates the carboxylic acid group. The methylprednisolone loaded nanoparticles (MP-loaded NPs) were prepared by a process of nanoprecipitation. The size and morphology of the MP-loaded NPs were characterized with transmission electron microscopyand (TEM) and dynamic light scattering (DLS). Subsequently, the efficiency of encapsulation and drug loading content were calculated. *in vitro* release of MP was evaluated in the phosphate buffer solution. The cytotoxicity of MP-loaded NPs against BV-2 cells were evaluated. The obtained ibuprofen nanoparticles have great application potentials in the treatment for SCI. and then, a rat model of SCI was used to compare the efficacy of controlled, nanoparticle-enabled delivery of MP with a single intravenous injection of MP-loaded NPs. The effects of the drug delivery system were evaluated on an acute SCI rat model by hindlimb motor function, histopathological evaluation, immunohistochemistry and the assessment of the level of the inflammatory cytokine (tumor necrosis factor alpha, TNF-α). Furthermore, ibuprofen was chosen as the biologically active carboxylic acids, which could surmount axon growth restrictions from myelin and proteoglycans and stimulate a significant neurite growth in the cultured dorsal root ganglion neurons [30]. It has a great potential in the synergetic effect treatment for spinal cord injury.

## RESULTS

### Synthesis of MP-loaded NPs

**Figure 1:**
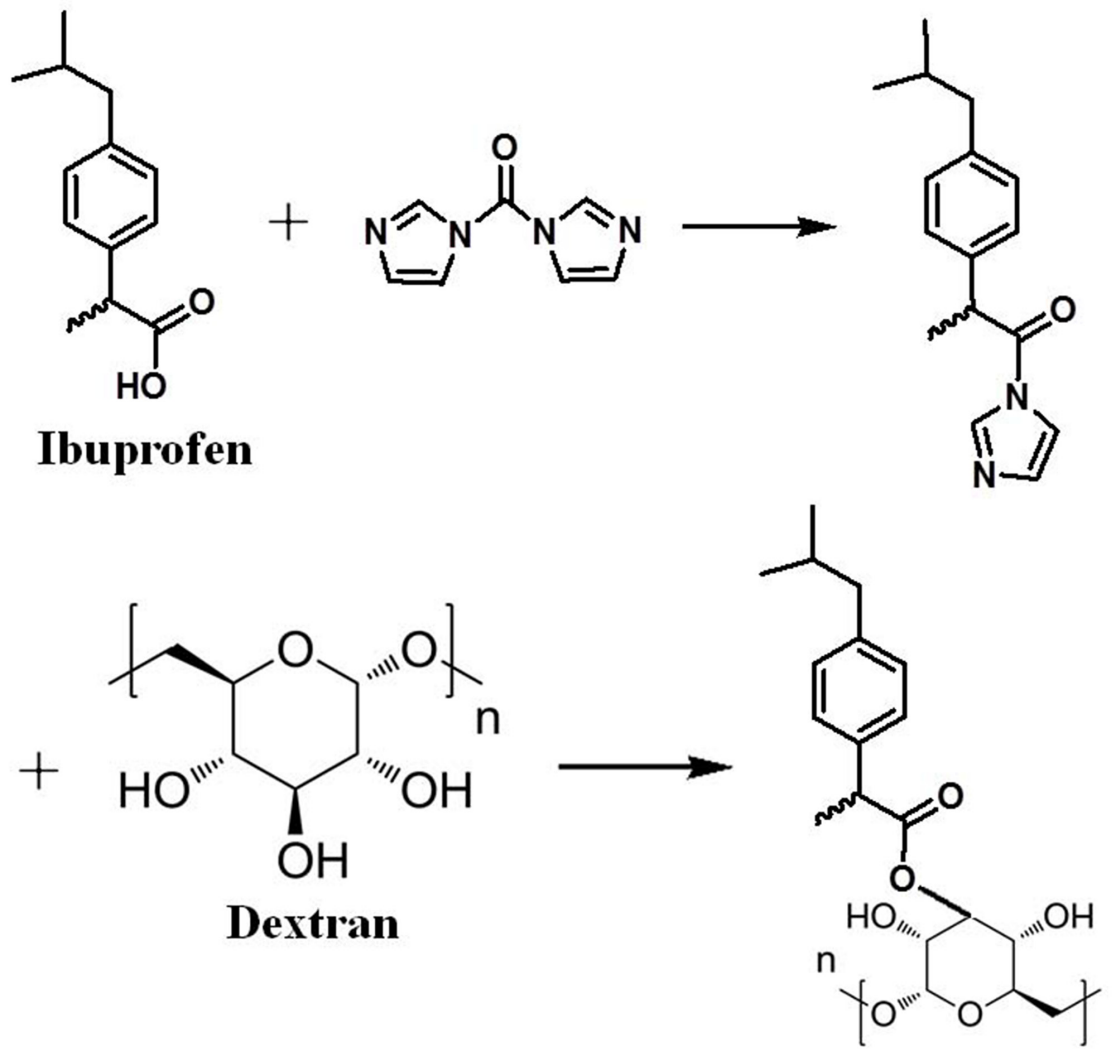
Synthesis of the MP loaded ibuprofen modified dextran based Nanoparticles

The synthetic route of MP-loaded NPs was summarized in figure (Figure 1). The way for the preparation of MP-loaded NPs is through a direct esterification linkage between the carboxylic acids of hydrophobic drug and the hydroxyl groups of the polymer backbone. The carboxylic acid with N, N-carbonyldiimidazole was activated in situ to achieve the effective esterification of Dextran. With the help of N, N-carbonyldiimidazole, the nanoparticle was prepared by a direct esterification linkage between the hydroxyl groups of Dextran and the carboxylic acid groups of ibuprofen. Soon afterwards, the polymer was added to the reaction solution and there is almost no depolymerization occurred.

### The characterations of MP-loaded NPs

**Figure 2:**
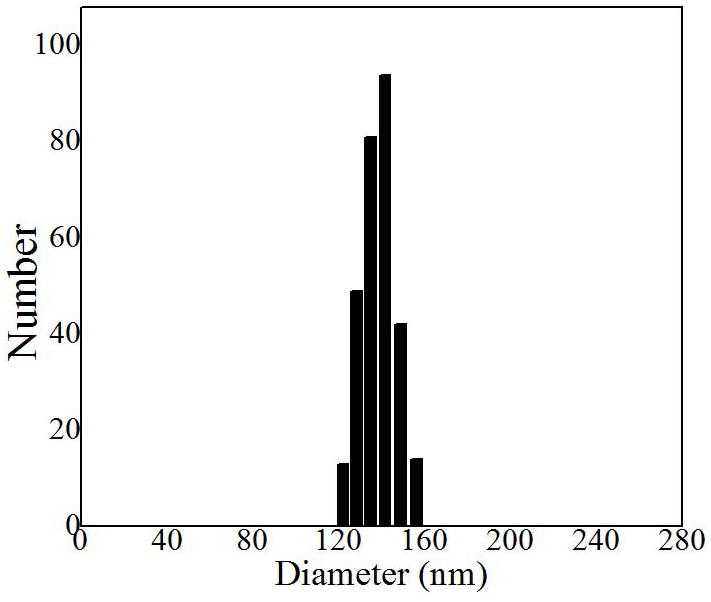
Hydrodynamic diameter distribution of MP loaded nanoparticles determined by DLS Nm, nanometer.

**Figure 3:**
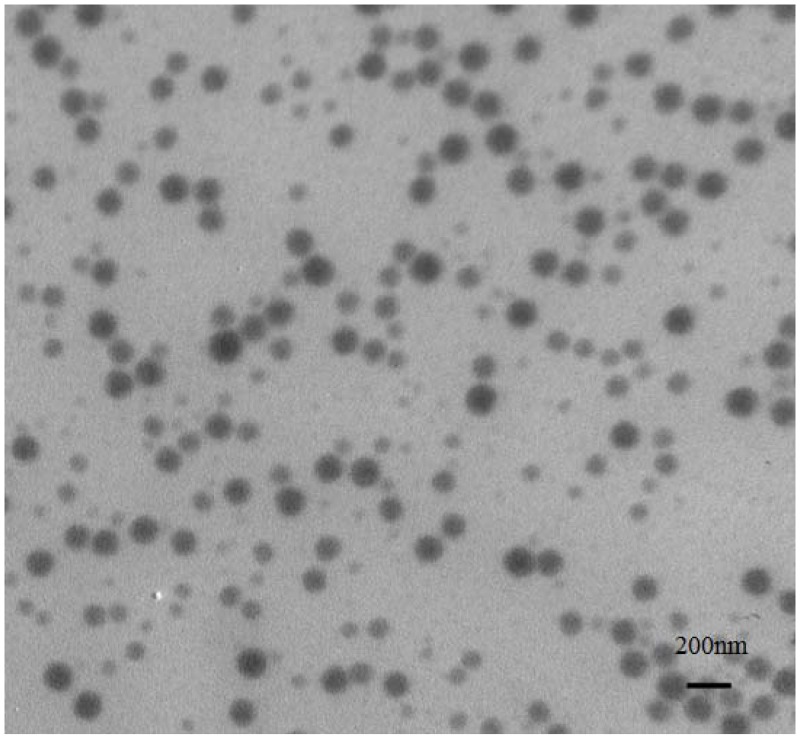
Typical TEM image of the MP loaded nanoparticles based on ibuprofen modified dextran

The characteration of MP-loaded NPs was shown in Figure 2 and Figure 3. The mean hydrodynamic diameter of MP-loaded NPs was determined to be 132 nm by DLS. It was obviously that the distribution of the size was relatively uniform. At the same time, the morphology of MP-loaded NPs were observed with TEM, the result was shown in Figure 3. It can be seen that MP-loaded NPs have a spherical shape while the diameter was determined to be about 127 nm with an uniform dispersion.

### Drug loading content and encapsulation efficiency of MP-loaded NPs

In order to assess the performance of the prepared nanoparticles in drug delivery, MP was selected and the MP-loaded NPs were prepared by a modified nanoprecipitation method. Then, the drug loading content and encapsulation efficiency of the MP-loaded NPs was measured. Taking advantage of the hydrophobicity of MP and the amphipathicity of ibuprofen modified Dextran based nanoparticles, MP and nanoparticles were dissolved in acetone solution together. Then, aqueous solution was added, and the nanoparticles were formed. The quantity of MP in the supernatant solution was measured as 0.03 mg after centrifugation, so the efficiency of encapsulation was calculated to be 97%. The centrifugal sedimentation weight 5 mg. After being dissolved in acetone, the quantity of MP in acetone solution was measured as 0.55 mg, so drug loading content of 11% was calculated. The MP-loaded NPs obtained under this condition with the relatively satisfactory efficiency and drug loading content, so, we chose to use this condition of the preparation of drug-loaded NPs in the following experiment.

### *In vitro* release profile of MP-loaded NPs

**Figure 4:**
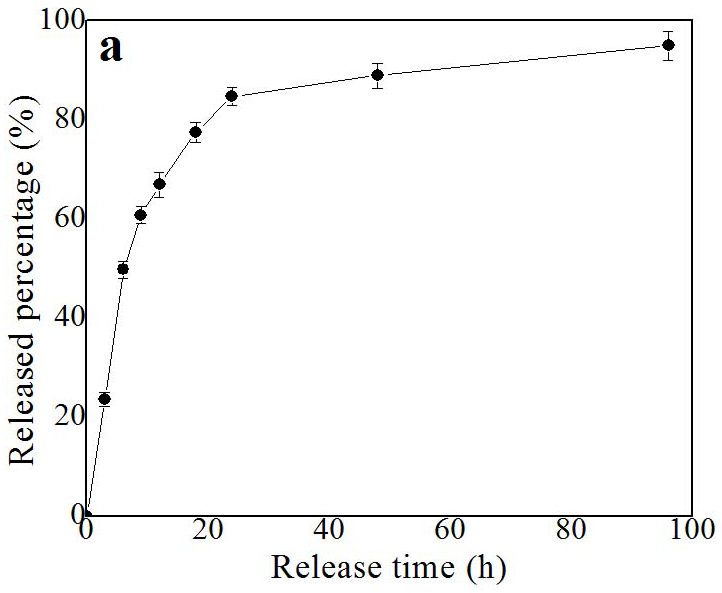
*In vitro* release profile of MP loaded nanoparticles in PBS medium at 37 °C Each point represents standard error. h, hour.

The *in vitro* release of MP from the of MP-loaded NPs was examined by using dialysis diffusion method. The *in vitro* MP release profile was shown in Figure 4 at 37 °C during 96 h of monitoring. About 94.5 % of the loaded MP is released from the MP-loaded NPs within 96 h.

### Cell viability test of MP-loaded NPs

**Figure 5:**
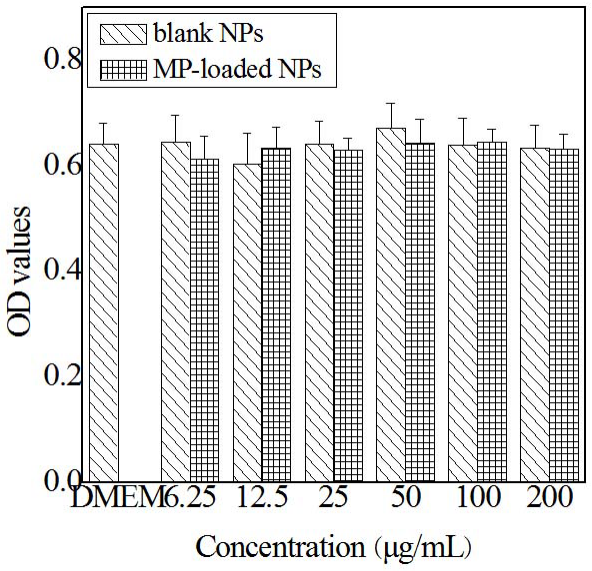
*In vitro* cytotoxicity of blank nanoparticles and MP loaded nanoparticles against BV-2 cells Blank NPs, blank nanoparticles. MP-loaded NPs, methylprednisolone loaded nanoparticles.

In order to examine the cytotoxicity of the MP-loaded NPs and blank NPs, the *in vitro* cytotoxicity test against the BV-2 microglial cells was carried out. The BV-2 microglial cells were cultivated in the DMEM culture medium and various concentrations of the MP-loaded NPs and blank NPs for 48 h (shown in Figure 5), correspondingly. It was found that there was no significant difference between the viability of BV-2 microglial cells cultured in the solution containing blank NPs or MP-loaded NPs and that in blank control of DMEM supplemented with 10 % fetal calf serum, which indicated that the blank NPs and the MP-loaded NPs have no obvious cytotoxic effect in all used conditions. Furthermore, it was found that the cells which were observed with optical microscopy after 12 h in culture dish in the solution were still active and all cells proliferated very well and maintained their normal configuration at all used conditions.

### Plasma concentration of MP

**Figure 6:**
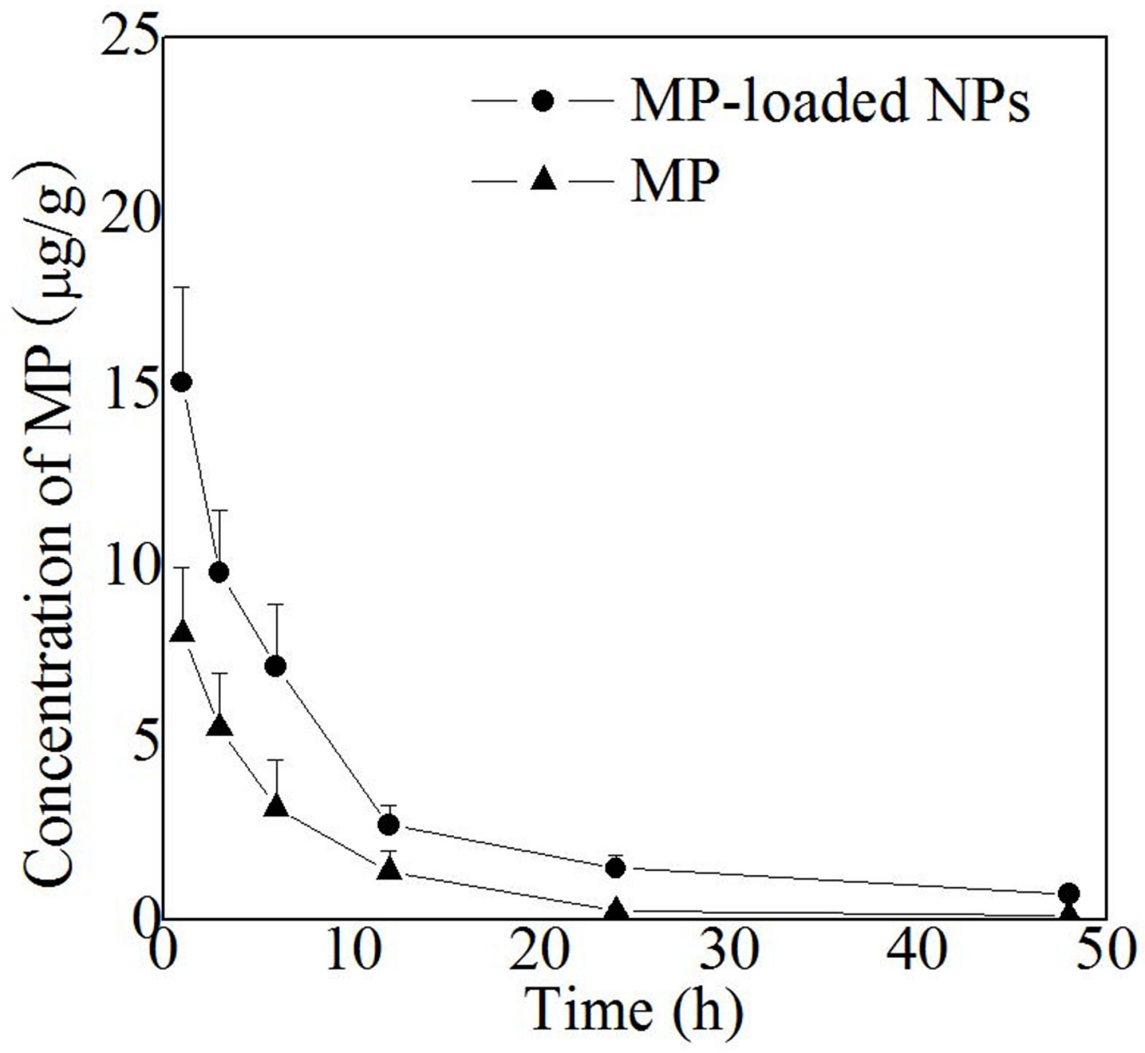
Plasma MP concentration of free MP and MP loaded nanoparticles in rats MP-loaded NPs, methylprednisolone loaded nanoparticles. MP, free methylprednisolone.

The blood plasma concentration of MP in rats was determined at various time points after the intraperitoneal injection of a single dose of reference formulation of MP or the MP-loaded NPs (equivalent 5 mg/kg MP). Then, the blood-drug concentration of MP was determined. As shown in Figure 6, at 1 hour post-injection, the blood plasma concentration of MP in the rats treated with the MP-loaded NPs was 15.20 ± 2.12 μg/g, while that in the rats treated with reference formulation of MP was only 8.29 ± 1.87 μg/g. In the extended period up to 48 hours post injection, the blood plasma concentration of MP in the rats treated with the MP-loaded NPs was 0.73 ± 0.08 μg/g, while that in the rats treated with reference formulation of MP was only 0.16 ± 0.11 μg/g. The plasma concentration of MP from the MP-loaded NPs group had increased significantly compared to the free MP group at every point post-injection which was obviously that the MP-loaded NPs had a relatively longer circulation, higher plasma concentration, and potentially better bioavailability.

### Results of neurological evaluation

Hindlimb motor function was evaluated by using the inclined plane test and modified Tarlov scoring system. *p* < 0.05 was considered significant.

**Figure 7:**
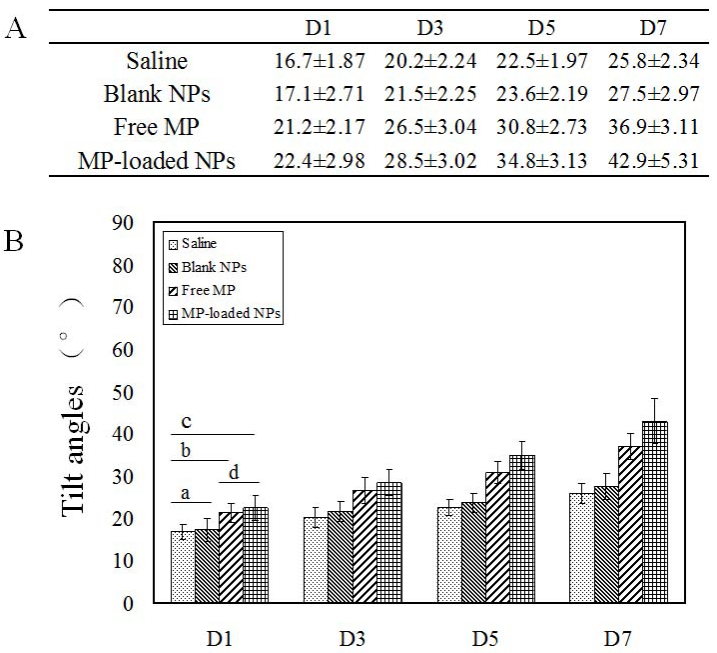
The tilt angles of the inclined plane test Blank NPs, blank nanoparticles. Free MP, free methylprednisolone. MP-loaded NPs, methylprednisolone loaded nanoparticles. D, day. **(A)** The results of the inclined plane test. **(B)** The tilt angles of the inclined plane test. ^a,D1,D3,D5,D7^ p > 0.05. ^b,D1,D3,D5,D7^ p < 0.05. ^c,D1,D3,D5,D7^ p < 0.05. ^d,D1^ p > 0.05. ^d,D3,D5,D7^ p < 0.05.

The results of the incline plate test were shown in Figure 7. There was not a significant difference between saline group and blank NPs group (^a,D1,D3,D5,D7^ *p* > 0.05). There was a significant improvement in tilt angles in free MP and MP-loaded NPs group compared to saline and blank NPs group (*p* < 0.05), at the same time, tilt angles of MP-loaded NPs group were significantly larger than that in free MP group (^d,D3,D5,D7^ *p* < 0.05). These results suggest that MP-loaded NPs is superior to free MP in terms of functional motor recovery following SCI.

**Figure 8:**
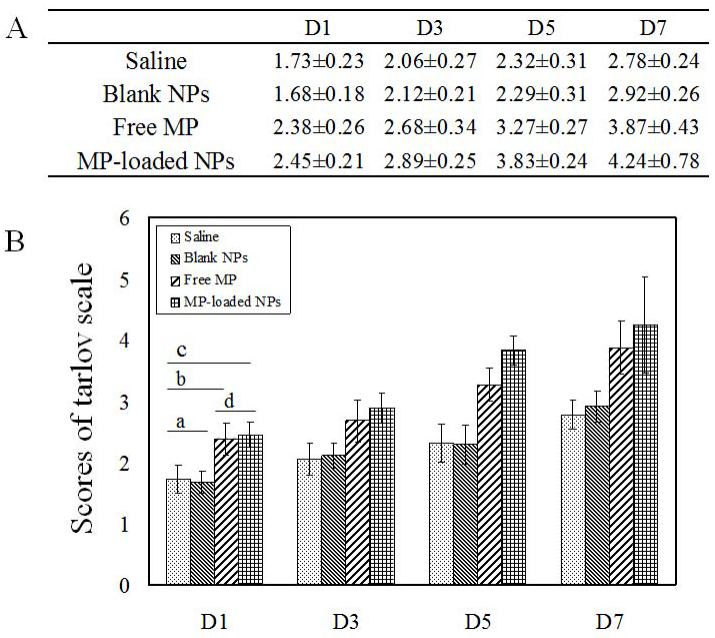
The scores of modified tarlov scoring system Blank NPs, blank nanoparticles. Free MP, free methylprednisolone. MP-loaded NPs, methylprednisolone loaded nanoparticles. D, day. **(A)** The results of modified tarlov scoring system. **(B)** The scores of modified tarlov scoring system. ^a,D1,D3,D5,D7^ p > 0.05. ^b,D1,D3,D5,D7^ p < 0.05. ^c,D1,D3,D5,D7^ p < 0.05. ^d,D1^ p > 0.05. ^d,D3,D5,D7^ p < 0.05.

The results of the modified Tarlov scoring system were shown in Figure 8, there was not a significant difference between saline group and blank NPs group (^a,D1,D3,D5,D7^ *p* > 0.05). There was a significant improvement in tarlov scores in free MP and MP-loaded NPs group compared to saline and blank NPs group (*p* < 0.05), at the same time, tarlov scores of MP-loaded NPs group were significantly higher than that in free MP group (^d,D3,D5,D7^ *p* < 0.05). These results indicated that free MP and MP-loaded NPs could promote the recovery of neurological deficits of spinal cord injury, while the recovery of nerve function in MP-loaded NPs group was significantly higher than that in free MP group.

### Results of histopathological evaluation

**Figure 9:**
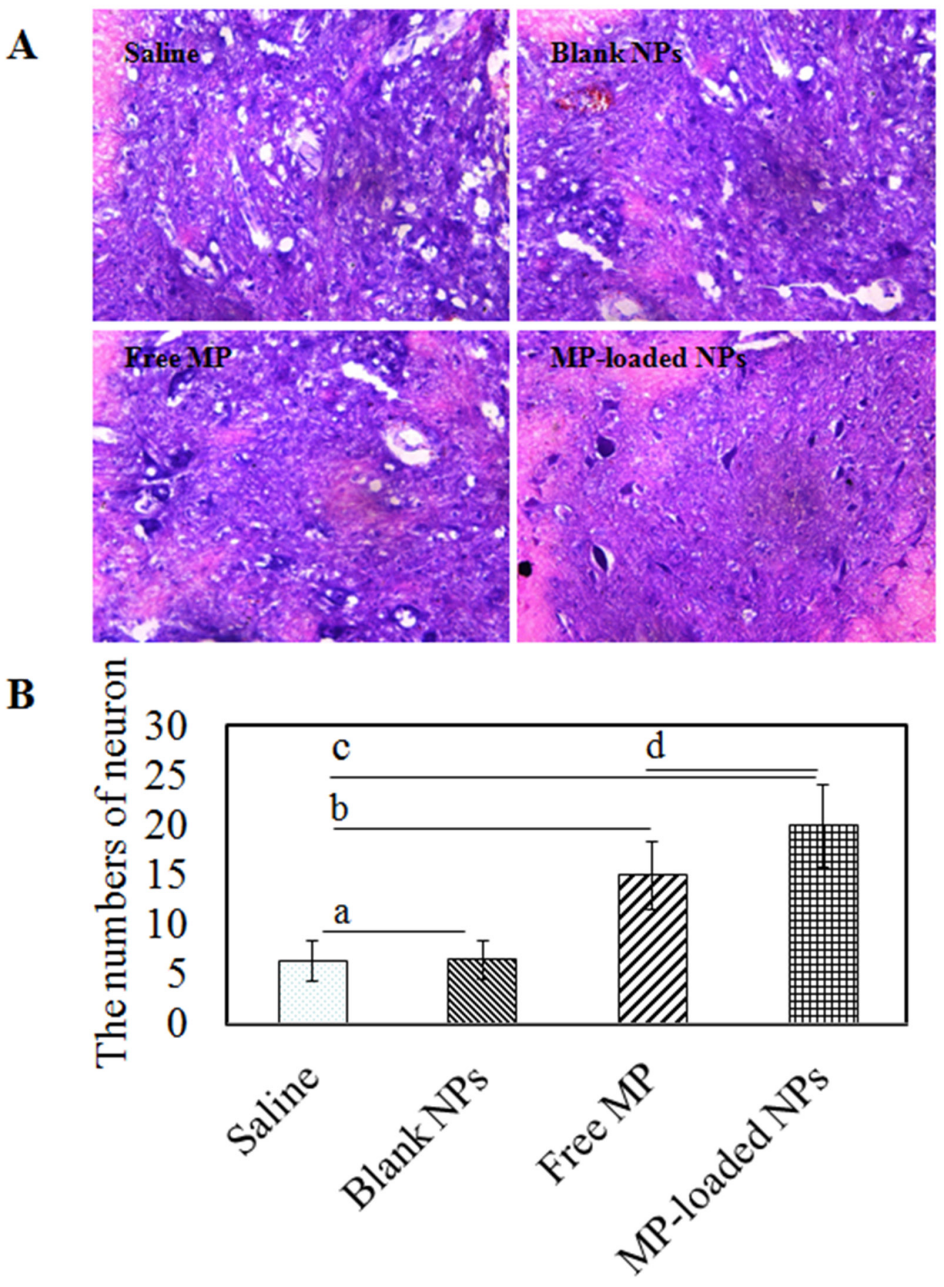
The histopathological evaluation of saline group, blank NPs group, free MP group and MP-loaded NPs group **(A)** HE staining results of SCI rats. **(B)** The numbers of neuron/mm^2^. ^a^ *p* > 0.05. ^b,c,d^ *p* < 0.05.

Histopathologically, saline group and blank NPs group showed normal morphology (shown in Figure 9). We observed the groups with optical microscopy, the main pathological changes are the nerve fibers edema, distorted, narrow, disintegration of the nerve membrane. The typical pathological changes of the impaired tissues after the spinal cord injury were hemorrhage, edema, necrosis, demyelination of axons, inflammatory cell infiltration, glial reaction and so on. By observing histological and pathological changes, we could find that in each group, the changes of demyelination of axons, necrosis, liquefaction, cavitation and gliocyte proliferation were obvious. But in saline and blank NPs group, the injuries were more serious with the worse clarity of the boundary between white and gray matter, the dispersed structure, small hemorrhagic foci. Therefore, the area of gray matter had partial dissolution and necrosis and the nerve fiber was highly edema. In free MP group, the clarity of the boundary between white and gray matter was just passable with active gliocyte proliferation and comparatively complete nerve cells. The denaturation and degree of necrosis was significantly alleviated compared to saline group and blank NPs group. Nevertheless compared with free MP group, MP-loaded NPs group had more numbers of nerve cells with integrity, significantly decreased degree of tissue edema and inflammatory cell infiltration and more active gliocyte proliferation. That was MP-loaded NPs could enhance growth of neurons and decrease degeneration of injuried neurons significantly. The administration of ibuprofen modified Dextran based nanoparticles following the spinal cord injury prevented and slowed neuronal degeneration, which could be attributed to its anti-inflammatory effect. Further studies were needed to evaluate effects of MP-loaded nanoparticles at different times and to elucidate the way and the mechanism of its action in injuries in the spinal cord.

### Results of TNF-α analysis

**Figure 10:**
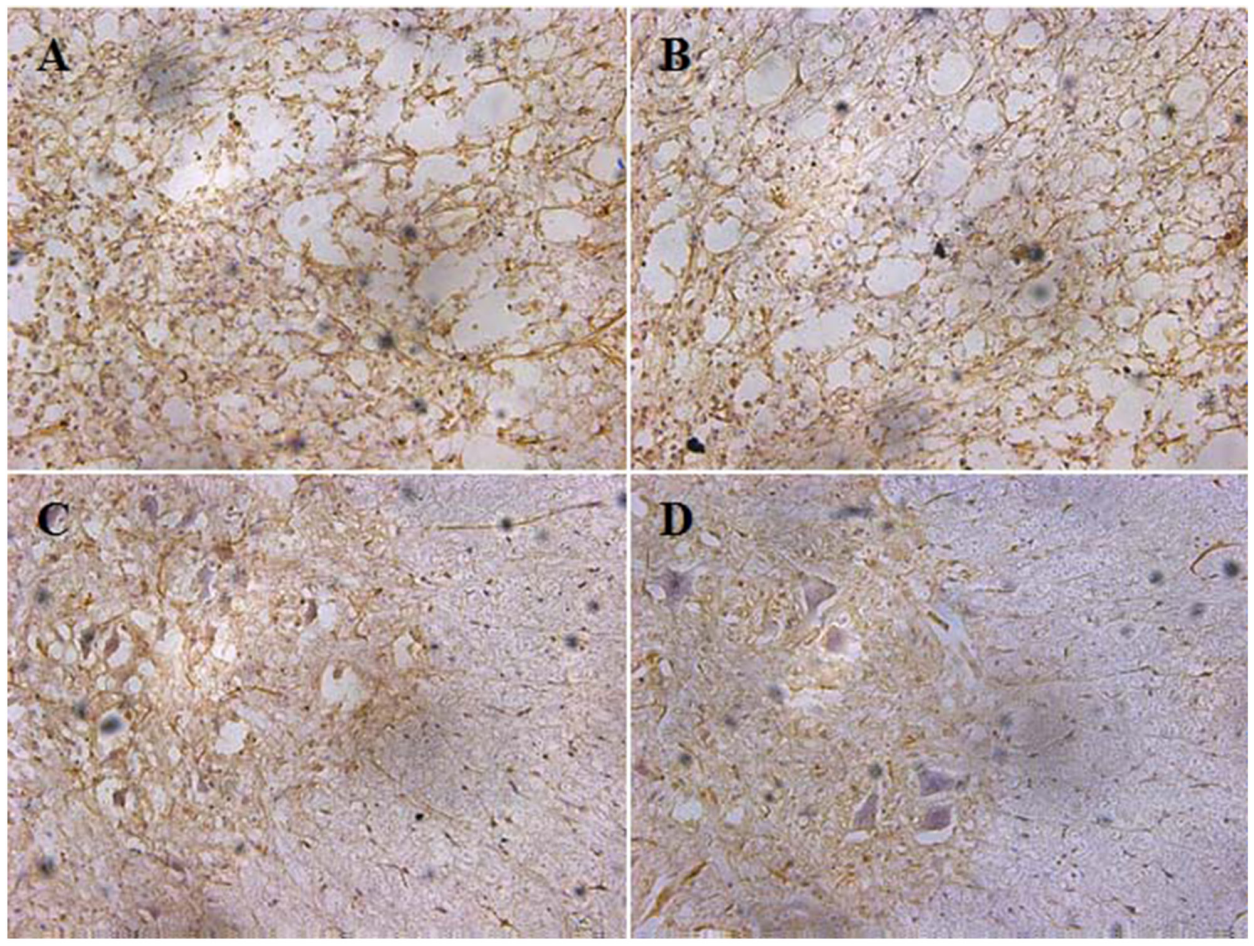
The tissure TNF-α levers of saline group, blank NPs group, free MP group and MP-loaded NPs group **(A)** saline group; **(B)** blank NPs group; **(C)** free MP group; **(D)** MP-loaded NPs group.

Immunostaining of TNF-α in the spinal tissue was detected as brown-yellow granules in Figure 10. There was a significant reduction in the tissue TNF-α levels in the free MP group and the MP-loaded NPs group. Additionally, the administration of MP-loaded NPs also showed significant reduction in the tissue TNF-α levels as compared with the free MP group. The difference in the TNF-α were not significant between the saline group and the blank NPs group. That was MP-loaded NPs could reduce the secretion of TNF-α significantly. The administration of MP loaded ibuprofen modified Dextran based nanoparticles following the spinal cord injury prevented and slowed neuronal degeneration, which could be attributed to its anti-inflammatory effect.

## DISCUSSION

Advancement in the field of the technology of nanoscience has alarmingly raised the call for comprehending the potential health effects caused by the exposure to nanoparticles based drug delivery system. Dextran, a branch of polysaccharide, has no surface charges and is often used to synthesize nanoparticles which leads to additional advantages for the drug-delivery system [18]. Because of its complete water-solubility and modified Dextran forming micelles, Dextran can be used to capture drug. As we all know, fructose unit chains were linked by glycosidic bonds and terminated at a single glucopyranoside ring, and the linear chains of fructose unit chains can form the polymer, which was the fundamental theory of the MP-loaded nanoparticles in our study [33]. On account of its nontoxicity, biodegradability, and hydrophilicity, it is a good candidate for biodegradable drug carriers, which would be a significant progress to prepare the copolymers of drug delivery system, and the copolymer based drug delivery system may combine both of their advantages [21, 22].

In our study, we report a method of synthesizing methylprednisolone loaded nanoparticles, specifically, the non-steroidal anti-inflammatory drug ibuprofen modified Dextran based nanoparticles. MP loaded nanoparticles were prepared by the self-assembly of ibuprofen modified Dextran with MP. After that, we studied the application for drug delivery in acute spinal cord injury. A possible way for the preparation of nanoparticles is through a direct esterification linkage between the hydroxyl groups of Dextran and the carboxylic acids of ibuprofen. The in situ activation of the carboxylic acid with N, N-carbonyldiimidazole could help to achieve the effective esterification of Dextran by avoiding the formation of acidic by-products during the reactions and the polymer being subsequently added to the solution with almost no degradation of polymer. In our work, it can be seen that MP-loaded NPs have a spherical shape with a homogeneous size. The mean hydrodynamic diameter of MP-loaded NPs was determined to be 132 nm by DLS. It can be seen that MP-loaded NPs have a spherical shape while the diameter was determined to be about 127 nm with an uniform dispersion observed with TEM. The diameter determined by TEM is smaller than that from DLS, which was probably caused by the shrinkage of the drug loaded nanoparticles in vacuum. The principle of the synthesis of the drug loaded nanoparticles was probablely that the ibuprofen segments form a core (the hydrophobic segments), while Dextran segments (hydrophilic segments) form a shell, and then, the MP (hydrophobic molecules) is encapsulated into the internal hydrophobic core of the nanoparticles. The efficiency of encapsulation of the MP-loaded NPs was calculated to be 97%, while drug loading content was calculated to be 11%, with the relatively satisfactory efficiency and drug loading content. The *in vitro* cytotoxicity test indicated that the MP-loaded NPs had no obvious cytotoxic effect in all used conditions. The plasma concentration of MP from the MP-loaded NPs group had increased significantly compared to the free MP group at every point post-injection which was obviously that the MP-loaded NPs had a relatively longer circulation, higher plasma concentration, and potentially better bioavailability. Hindlimb motor function was evaluated by using the inclined plane test and modified Tarlov scoring system. These results indicated that free MP and MP-loaded NPs could promote the recovery of neurological deficits of spinal cord injury, while the recovery of nerve function in MP-loaded NPs group was significantly higher than that in free MP group. Results of histopathological evaluation showed that MP-loaded NPs could enhance growth of neurons and decrease degeneration of injuried neurons significantly. Results of TNF-α analysis showed that the administration of MP-loaded NPs could significantly reduced the tissue TNF-α levels as compared with the free MP group.

During our experiments, we can obtain the stabilized methylprednisolone loaded ibuprofen modified Dextran based nanoparticles. As drug carriers, the MP-loaded NPs have the slow-release property, which have better functions of stimulating nerve growth, inhibiting the release of inflammatory factor (TNF-α), reducing the loss of nerve cell and promoting the recovery of neural function, especially reducing the toxicity caused by clinical high dose of MP after spinal cord injury.

The applications of nanoparticle based drug delivery system in spinal cord injury remain in the stage of *in vitro* and animal researches, while the clinical applications of nanoparticle based drug delivery system haven’t been seen in spinal cord injury, so in-depth studies and a large number of human trials are needed to prove it.

As our work proceeded, our understanding of nanoparticle based drug delivery system is further deepened, on the basis of present work, the related work can also be an in-depth study and the exploration in the following aspect. In further studies, we can try to use different biocompatible and biodegradable materials to prepare the different materials with different size, different surface properties and stability of nanoparticle based drug delivery system to meet the needs of screening the best drug carrier for spinal cord injury.

Nanoparticle based drug delivery system, as a new type of pharmaceutical preparations, has got rapid progress. With the development of science and technology, we will be bound to prepare more intelligent, more sophisticated nanoparticle based drug delivery system, so as to realize the automatic target, timing and quantitative release of drugs with the reduced side effects is urgently needed to be studied.

We firmly believe that with the development of science and technology, nanoparticles based drug delivery system will become a powerful weapon of human conquest of disease.

## MATERIALS AND METHODS

### Synthesis of ibuprofen modified dextran based nanoparticles

First, 0.5 g (2.42 mmol) ibuprofen[2-(4-isobutylphenyl) propanoic acid), Acros Organics Company, the United States] were added to a 5 mL Dimethyl sulfoxide (DMSO, Gibco company, the United States), then, 0.43 g (2.66 mmol) of N, N’-carbonyldiimidazole (Shanghai Sigma-Aldrich Company, China) were added to the solution and the reaction mixture was stirred at room temperature for 1 hour. 0.5 g (3.1 mmol, the fructose unit) of Dextran (Shanghai Alladin Reagent Company, China) was subsequently added to the mixture and the reaction was allowed to react for 24 hours at 80 °C under stirring. The resultant solution was precipitated into 100 mL cold water. The precipitates were filtered out, washed several times with water and dried under vacuum to obtain Dextran-ibprofen conjugates.

### Preparation of methylprednisolone-loaded nanoparticles (MP-loaded NPs)

MP-loaded NPs were prepared by the self-assembly of MP (Yueyang Huanyu Pharmaceutical Company, China) and the ibuprofen modified Dextran copolymer. Together with 1 mg of MP, 5 mg of ibuprofen modified Dextran based nanoparticles were dissolved in 1 mL of acetone, and then, the mixture was dropped into 10 mL of hot water (50 °C) under a slow stirring. Later on, under a reduced pressure at room temperature, the acetone was removed. Subsequently, a cellulose acetate filter with the average pore size of 0.22 μm was used to filter this suspension to remove the unloaded MP. And then, MP-loaded NPs were obtained.

### Characterization of MP-loaded NPs

A Brookheaven BI9000AT dynamic light scattering system (DLS, Brookheaven Instruments Corporation, the United States) and a LSM-710 laser scanning confocal microscope (ZEISS, Germany) was used to measure the size of the MP-loaded NPs. The incident angle was 90 degrees, and the incident wavelength of the laser was 633 nm. Each sample were measured three times, 60 times per scan. The concentrations of the samples were measured at 0.05%. The test temperature was room temperature, and the average diameter of the particle was presented in Mean ± SD. The pH value of the solution was adjusted by using sodium hydroxide and hydrochloric acid. Transmission electron microscope (TEM, JEM-1010, Japan) analyses were performed after the sample being stained with a phosphotungstic acid solution (2 %, w/v).

### Drug loading content and encapsulation efficiency

The prepared acid of MP-loaded NPs was centrifuged at high speed (30000rmp/min), dried out, and the general quantity was determinated. Then, it was dissolved in 1mL of methanol. Using a pre-determined calibration curve, the concentration of MP in the resulting methanol solution was determined by high performance liquid chromatography (HPLC). Equipped with a C-18 Wondasil-HPLC analysis column and a Shimadzu UV detector, HPLC analysis was performed on a Shimadzu LC-15A HPLC system (Shimazu, Japan). The mobile phase consisted of a mixture of acetonitrile:water (v/v, 34:66, pH = 3.4), and delivered at a flow of 1.0 mL/min at 25 °C. The lyophilized nanoparticles were accurately weighted before dispersing in methanol. The drug loading content and encapsulation efficiency was respectively calculated by the following formulas:

$$\text{Drug loading content}\%=\frac{\text{Weight of the drug in the nanoparticles}}{\text{Weight of the nanoparticles}}\times 100\%$$

$$\text{Encapsulation efficiency}\%=\frac{\text{Weight of drug in the nanoparticles}}{\text{Weight of the feeding drug}}\times 100\%$$

### *In vitro* drug-release profiles of the MP-loaded NPs

A 15 kDa MWCO membrane was used to dialyze the aqueous suspension of the MP-loaded NPs containing 150 μg of MP with 10 mL of phosphate buffer solution (PBS) at 37 °C. At a specific time point (0, 3, 6, 9, 12, 18, 24, 48, and 96 hours), a sample (1 ml) of aliquot was taken out from the bulk PBS solution and then the same volume (1 ml) of medium was added to the suspension. HPLC with a pre-established calibration curve was used to measure the released concentrations of MP in the sample medium. At each time point, the accumulative release percentage was calculated. And the measurements were repeated three times and the average of three measurements was shown as the results.

### *In vitro* cytotoxicity

A MTT (3-(4, 5-dimethylthiazol-2-yl)-2, 5-diphenyltetrazolium bromide) assay was used to test the cytotoxicity of samples. BV-2 cells were grown in a Dulbecco’s modified Eagle’s medium (DMEM) containing 4 mM glutamine, 100 μg/mL streptomycin, and 100 U/mL penicillin with 10 % fetal bovine serum in a 5 % CO2 atmosphere at 37 °C. And then, the cells, at a density of 5000 cells per well, were seeded into a 96-welled plate, and incubated with 100 μL of culture medium containing different doses of samples at 37 °C for 2 days. After being incubated, the media in each well were removed and PBS was used to wash the cell three times. And then, MTT solution (5 mg/mL, 10 μL) was added to each well and cultivated for 4 hours. The supernatant liquid was discarded, after that, 100 μL of DMSO (Dimethyl Sulphoxide) was added. A microplate reader (Safire, Tecan, Swiss) was used to observe the OD values of plates at 570 nm. Without any modifications after various treatments, the results were expressed as the percentage of cells relatively to the control cells.

### Determination of the blood-drug concentration *in vivo*

The study protocol was approved by ethics committee of Affiliated Hospital of Nantong University. Healthy, specific pathogen free, male Sprague Dawley rats (N = 40; 6-8-weeks-old; weight 180-220 g) were purchased from Experimental Animal Center of Nantong University. Eight rats were randomly selected from all the rats, after anesthetization by pelltobarbitalum natricum (30 mg/kg) with intraperitoneal injection, they were randomly divided into two groups, the MP-loaded NPs and MP groups. In MP group, MP (80mg/kg) was injected within 15 minutes by intravenous injection, of which the same dose of MP was injected in MP-loaded NPs group. Using a pre-determined calibration curve, the blood-drug concentration of MP was determined by HPLC, at a specific time point (2, 4, 6, 12, 24, and 48 hours). And the measurements were repeated three times and the average of three measurements was shown as the results.

### The establishment of acute SCI of SD rats

Modified Allen’s weight-drop technique was used in this study. Briefly, spinal cord of the left 32 SD rats (Sprague-Dawley rats) was moderately injured under general anesthesia after anesthetization by pelltobarbitalum natricum (30 mg/kg) with intraperitoneal injection. Hair of the rats was shaved, and by using the eighth thoracic vertebra as a marker, the vertebral plates were removed through midline incisions on the back to expose the chest 7-11 of the spinal cord, maintaining an intact dura. Acute SCI (ASCI) was induced by a 1.2-mm-diameter metal cylinder weighing 12 g, dropping from a height of 3.0 cm in a vertical plastic tube directly onto the exposed spinal cord, which resulted in acute SCI with an injury force of 36 g·cm. The occurrence of the emergences including tail wagging reflexes, lower limbs and body retraction flutter, and flaccid paralysis of the lower limbs indicated the efficiency of the model. After the interventions, the overlying back muscles and the skin, in layers, were sutured. Drug interventions for the first time were taken after the model of acute SCI was successfully built within 15 minutes by intravenous injection. To prevent infection, gentamicin (3 mg/kg) was used by intramuscular injection daily in the first three postoperative days. And manually artificial assistant of defecating urine was taken every three hours per day until automatic bladder function resumed.

### Application of MP-loaded NPs

The rats with ASCI were randomly divided into the following four groups (8 rats in each group): MP-loaded NPs group, free MP group, blank NPs group, and saline group. After the model of ACSI was successfully built, drug interventions were taken within 15 minutes by intraperitoneal injection for the first time. Intravenous injection of MP (80mg/kg) was initiated 15 minutes post-operation in free MP group, of which the same dose was injected in MP-loaded NPs group. While in blank NPs group, the same dose of nanoparticals was injected as in the MP-loaded NPs group. In saline group, postoperative intravenous physiological injections of normal saline (0.9% NaCl) were administered with equivalent dose in free MP group with the same volume, and all models were sampled within the scheduled time. Any dead rats were replenished randomly.

### Evaluation of hindlimb motor function

1, 3, 5, and 7 days postoperatively, eight rats from each group were randomly selected for evaluation of hindlimb motor function by using the inclined plane test [31] and modified Tarlov scoring system [32].

In the inclined plane test, the rats were horizontally placed on a smooth wooden platform. From the horizontal position (0°), every 5 seconds, one side of the platform was elevated by 5°. The maximum angle at which the rat was able to stay on the board for a minimum of 5 seconds was taken as the value for hindlimb motor function of that rat.

The modified tarlov scoring system was used to assess the neurological functions at the 1, 3, 5, and 7 day after the surgery. Locomotion of the rats was scored as follows: score 0, no movement of the lower limbs and spastic paraplegia; score 1, slight movement of the lower limbs and spastic paraplegia; score 2, good movement of the lower limbs, but still inability to stand; score 3, ability to stand but inability to walk; score 4, ability to walk, but can not lasting; score 5, ability to walk, but inability to run; score 6, complete recovery of hind-limb function.

### Histopathological evaluation

Seven days after surgery, four rats from each group were selected at random, after anesthesia with 10% chloral hydrate (350 mg/kg), thoracotomy was performed and intubated from the left ventricle to the ascending aorta through the chest incision. And then, the heart was perfused. The right atrium was cut open and 200 mL of physiological saline was perfused at a constant rate. And 4% polyformaldehyde was perfused. When the fluid flowing from the right atrium became colorless, a 1.5 cm length of spinal cord including the injured tissue was harvested. All this was used for hematoxylin-eosin staining to determine the degree of injury. Each cord segment was immersed in 4% formaldehyde for 72 h. After fixation spinal cord tissues were dehydrated with graded alcohol series and embedded in paraffin. After obtaining tissue blocks, 5 m thick transverse sections were cut, stained with hematoxylin and eosin (H&E), and viewed under an optical microscope (Olympus, Tokyo, Japan) to study the congestion, hemorrhage, edema, necrosis, and neuronal viability. The complete neuron numbers were counted at 5 randomly selected fields per sample.

### Tissue TNF-α analysis

The TNF-α level in the spinal tissue was measured using immunohistochemistry. The sections were subsequently used for immunohistochemical analysis as described below. The spinal cord sections were first washed (3 × 10 min) in PBS, then it was incubated in blocking solution including 3% normal goat serum and 0.1% Triton X-100 in TBS for 30 minutes at the room temperature. And then, sections were incubated in a rabbit polyclonal anti-TNF-α antibody (1:100; ab6671, Abcam, Cambridge, United Kingdom) in blocking solution overnight at 4°C. At the following day, all slides were washed in cold PBS, following which they were incubated in the goat polyclonal secondary anti-rabbit antibodies (1:1000; ab150077, Abcam, Cambridge, United Kingdom) prepared in blocking solution at room temperature for 1 hour. After that, sections were washed in PBS three times, 10 minutes per time. Finally, the slides were incubated in 3,3-diaminobenzidine for 3 minutes, and then they underwent Mayer’s hematoxylin counterstaining for 30 seconds and were mounted. The positive neuron numbers of TNF-α were counted at 5 randomly selected fields per sample, for which the histology and immunohistochemistry was performed simultaneously in all spinal cord samples.

### Statistical analysis

SPSS 19.0 statistical software (SPSS Inc., Chicago, IL, USA) was used for data analysis. Counting data comparisons between groups were subjected to the χ2 test. And of at least three independent experiments, all the results were expressed as the mean ± S.D. For all statistical analyses, P values < 0.05 were considered to be statistically significant.

## Abbreviations

(MP): Methylprednisolone
(NPs): Nanoparticles
(SCI): Spinal cord injury
(TEM): Transmission electron microscopy
(DLS): Dynamic light scattering
(MP-loaded NPs): Methylprednisolone loaded nanoparticles
(TNF-α) levels: Tumor necrosis factor alpha
(HPLC): High performance liquid chromatography
(DMEM): Dulbecco’s modified Eagle’s medium
(Dimethyl Sulphoxide): DMSO
(Sprague-Dawley rats): SD rats
(ASCI): Acute spinal cord injury
(Fig.).: Figure
